# Ogeechee Medical Association—Stated By-Monthly Meeting

**Published:** 1888-07

**Authors:** R. Y. Lane

**Affiliations:** Secretary


### OGEECHEE MEDICAL ASSOCIATION—STATED BI-MONTHLY MEETING.

REPORTED BY R. Y. LANE, SECRETARY, MARCH 8, 1888.

The President, E. W. Lane, M. D., in the chair.

Dr. Daniel E. Gray, having been called upon at a previous meeting for his experience in the use of "Mistletoe" as an oxytocic, read as follows:

Mistletoe is a parasitic plant, growing on various trees in this country, principally oaks, gums, persimmon, apple and plum trees, as well as on many other kinds of trees, and I have no doubt the most of you are familiar with the plant. It is claimed by some authors to have antispasmodic properties. My attention was first called to its use as an oxytocic by my esteemed friend C. L. Sample, M. D., who had seen it used by the stock raiser, to relieve a cow of the secundines. I prepared a saturated tincture of the green leaves, and used it as follows: Mrs. P., mother of five children, the last of which she had been delivered by a midwife, ten hours before I saw her. I found Mrs. P. very much excited, as the midwife in her attempt to deliver the placenta had torn off the cord from the placenta, while it remained yet in the uterus, and held there by hour-glass contraction. I deemed this a good case to try my tincture of mistletoe. Accordingly I gave the patient 15 gtts. of the tincture every ten minutes, and watched the effect. After the second dose, the volume of the pulse seemed increased; after the third dose, the spiral or hour-glass contraction changed to natural, and soon after the fourth dose, she had a sharp, expulsive pain, and the placenta was expelled.

My second case was that of a lady with her first child, who had miscarried at the fifth month of her pregnancy. She had been delivered about two hours before my arrival. I found that the cord had been broken off and the lady was having profuse hemorrhage. I gave 20 drops of mistletoe tincture, and applied cold wet cloths to abdomen; continued the dose every fifteen minutes; after the third dose, I had the satisfaction of seeing my patient relieved of the contents of the uterus, and the hemorrhage ceased.

Third case was that of a negro woman, at about the fifth month of her pregnancy. The fœtus had been dead sometime, she was attended by a negro midwife, who could not deliver the patient, and had me sent for. I gave her 20 drops of the tincture, every fifteen minutes, until she had taken five doses, when the contents of the uterus were expelled in mass. It was a mass of rotten matter, and emitted a most horrid and offensive smell. There was not a particle of hemorrhage.

My next case was that of a lady who had a tedious labor, though otherwise natural. Shortly after being put to bed she was attacked with a severe hemorrhage. I treated it in the usual way, giving ergot, applying wet cold cloths to the abdomen, etc., and nothing I did seemed to control it; after everything else I could think of had failed, I gave ½ drachm dose of tincture of mistletoe, and in fifteen or twenty minutes the uterus contracted, expelling clots, etc., and the hemorrhage ceased.

My next case was that of a lady aged thirty-one years, the mother of five children. I found her very much debilitated, had been in bad health for sometime, her menses very irregular, showing itself every few days, and sometimes amounting to a considerable hemorrhage. When I saw her she was flowing profusely, and was so weak she could not be raised up without fainting. I gave her ½-teaspoonful doses tincture mistletoe every fifteen minutes until she had taken about six doses, when the hemorrhage ceased entirely. I put her on tinct. mur. iron as a tonic, and she made a good recovery.

I have used it many times in my labor cases, especially where the pains are of a cramp-like nature, rather than expulsive, as I am convinced that it acts upon the longitudinal masses of the uterus.

Dr. E. A. Perkins said that his attention was called to the use of the mistletoe as an oxytocic, several years ago, by an overseer, on a plantation on which he did a large practice. He had noticed that nearly all the negro women on the plantation were suffering with endometritis, and called the attention of the overseer to the fact. The overseer remarked (pointing to some mistletoe that grew on some oaks in the negro quarters), "that is what has caused it. They take it to keep from having babies."

Dr. J. W. Johnson said that he had not used the drug himself, but had no doubt of its efficacy as an oxytocic, from what he had learned from others of the profession, and that he was much interested in that case reported by Dr. Gray, in which he used it in conjunction with ergot. As ergot is known to act on the spiral muscles, and if it is true that mistletoe acts on the longitudinal, he had no doubt that a combination would be useful in many cases.

Dr. E. W. Lane said that he had used the mistletoe with happy results in many cases, and he had no doubt that it acted on the fundus of the uterus. He had used it that grew on the oak, that grew upon the blackgum, and that which grew on the persimmon; and his observation was, that that which grew upon the persimmon acted most promptly, and that which grew on the oak less so, or in other words, that it acted quicker, when given in same dose. He asked the members present if it might not be that the tree it subsisted on had something to do with therapeutical effect.

Dr. J. W. Johnson said it is highly probable that it did, as it is admitted generally, that the land on which a plant grew, influenced its therapeutical action.

It being the only business before the Association at this time it adjourned to meet again on the second Thursday in May next.

R. T. LANE, M. D., *Secretary.*

